# Reflective Properties of a Polymer Micro-Transducer for an Optical Fiber Refractive Index Sensor

**DOI:** 10.3390/s20236964

**Published:** 2020-12-05

**Authors:** Paweł Marć, Monika Żuchowska, Leszek R. Jaroszewicz

**Affiliations:** Faculty of Advanced Technologies and Chemistry, Military University of Technology, 00-908 Warsaw, Poland; monika.zuchowska@wat.edu.pl (M.Ż.); jarosz@wat.edu.pl (L.R.J.)

**Keywords:** optical fiber refractive index sensor, photopolymerization, polymer microtip

## Abstract

A polymer microtip manufactured at the end of a multi-mode optical fiber by using the photopolymerization process offers good reflective properties, therefore, it is applicable as an optical fiber sensor micro-transducer. The reflective properties of this microelement depend on the monomer mixture used, optical fiber type, and light source initiating polymerization. Experimental results have shown that a proper selection of these parameters has allowed the design of a new class of sensing structure which is sensitive to the refractive index (RI) changes of a liquid medium surrounding the microtip. An optical backscatter reflectometer was applied to test a group of micro-transducers. They were manufactured from two monomer mixtures on three different types of multi-mode optical fibers. They were polymerized by means of three optical light sources. Selected micro-transducers with optimal geometries were immersed in reference liquids with a known RI within the range of 1.3–1.7. For a few sensors, the linear dependences of return loss and RI have been found. The highest sensitivity was of around 208 dB/RIU with dynamic 32 dB within the range of 1.35–1.48. Sensing characteristics have minima close to RI of a polymer microelement, therefore, changing its RI can give the possibility to tune sensing properties of this type of sensor.

## 1. Introduction

Feasibility of the polymer microelement manufactured at the end of an optical fiber was previously demonstrated in microscopy [1], as well as fiber to fiber connector technology [2]. The author’s research has shown the possibility to shape the geometry of such microstructures by using selected types of optical fibers, chemical compounds, and by tuning parameters of the photopolymerization process [3]. Moreover, the application potential of this type of optical element as an optical fiber sensor micro-transducer was also mentioned [4,5,6]. Therefore, in this paper, an extended description of a polymer microtip application as an optical fiber refractive index (RI) sensor was reported.

Optical fiber technology dedicated to RI sensors has been evolving since its early stage. It offers new set-ups and implementations in various research fields. The common configurations used are mainly based on long-period gratings [7,8,9,10,11], Bragg gratings [12,13,14], tapered fiber [15,16,17], modal interferometers [18,19,20]. Additionally, in some applications, sensitivity and selectivity enhancement need a specially synthesized thin absorbent layer able to attract specific chemical or biological agents [21,22,23,24]. The mentioned above sensors allow measuring the change of RI from an optical transmission signal. However, the sensor configuration reflective type is also possible. In this configuration, it is possible to use various types of transducers, and input and reflected light interfere with what allows calculation of RI changes. The transducer is a set of simple single-mode fibers with reference liquid [25], core-less fiber with a gold reflector [26], tapered twin-core fiber [27], a cascade of a double-core fiber [28], or single-mode fiber transformed into an ellipsoidal shape [14] with a Bragg grating. Basic principles of work of these sensors are intensity modulation measurements caused by changes of Fresnel reflection [25], interference amplitude of input and back-reflected light [26,27], and reflectivity of a single-mode fiber core mode [14,28]. In our paper, the polymer microtip formed at the end of an optical fiber operating in a reflective mode as was shown by some examples [4,5,6] was reported. 

The microtip manufacturing process is based on a photopolymerization phenomenon and is well described in the literature [3,4,5,6,29,30,31]. This method has many advantages, such as low cost, low energy consumption, fast polymerization chain reaction, working at ambient temperature, and the possibility of creating 3D structures with required geometry and micrometric sizes. Most of these optical microelements were fabricated on various single-mode optical fibers [4,29,30,31,32]. However, polymer microtips on multi-mode optical fibers (MMFs) have better sensing properties [3,4,5,6,33].

This paper is divided into three sections. The first one presents a description of the optimization of manufacturing technology and the second one contains the experimental results. Both are summarized in the third one—the discussion section.

## 2. Manufacturing Technology of a Polymer Micro-Transducer

### 2.1. Photopolymerization Process and Monomer Mixtures

The manufacturing technology of the polymer microtip is based on a photopolymerization phenomenon. Light with a selected spectrum is absorbed in a monomer mixture and initiates a free radical polymerization. Depending on the mixture composition, including the type of initiator, different light sources from the UV-VIS spectrum range are used to start this process.

After testing various mixtures based on different multifunctional acrylate monomers, two materials were finally selected: 3-functional pentaerythritol triacrylate (PETA, Sigma-Aldrich, St. Louis, MO, USA) and 2-functional tricyclo decanedimethanol diacrylate (TCDMA, Sigma-Aldrich, St. Louis, MO, USA). The final mixture consists only of a monomer and a sensitizer—when it is cured with UV light. When the VIS light source is used, it requires an additional compound as a co-initiator. In the first mixture (UV mixture) as a sensitizer was used 2,2-Dimethoxy-2-phenylacetophenone (DMPAP) [3,6,33]. In the second mixture (VIS mixture), the Eosin Y disodium salt as a sensitizer and methyldiethanolamine (MDEA) as a co-initiator were used [3,4,5,6,32]. The possible percentage compositions of mixtures may differ. Monomer content in a UV mixture can be within the range of 99.70–99.95%, whereas for a VIS mixture from 89.00 to 92.40%. In the latter case, the co-initiator weight varies between 7.0–10.0% and the rest weight percentage belongs to the sensitizer. Manufactured microtips within the ranges of the above-mentioned percentage compositions maintained similar optical properties.

### 2.2. Technology and Optimization of a Polymer Micro-Transducer

The manufacturing process of the polymer microtip consists of several steps. First, a cleaved optical fiber is placed in a holder and a liquid monomer mixture drop is applied. Then, the drop is illuminated by a light beam, launched from the other end of the used optical fiber and a polymer microtip is formed. The unpolymerized part of the drop is washed by alcohol and dried. 

The geometry of polymer microtips depends on several parameters. The most important are mixture composition and close related spectral characteristics of a light source, amount of delivered energy (exposure time and optical power), and optical fiber type [3]. However, the application of this micro-element as a sensor transducer requires extended studies of the influence of these parameters on microtip reflective properties. 

The selection of two monomer mixtures has imposed testing different light sources to initiate the photopolymerization. The equipment used were solid-state laser emitting at a wavelength of 532 nm (Samba, Cobolt AB, Solna, Sweden)—named LASER 532 and two fiber-coupled LEDs: UV with a central wavelength of 365 nm (M365F1, Thorlabs, Newton, NJ, USA)—named UV LED and VIS with a central wavelength of 512 nm (M530F2, Thorlabs, Newton, NJ, USA)—named VIS LED. Spectral characteristics of the mentioned sources were described in detail in [3]. 

Moreover, in experiments, three types of MMFs were used. The main differences between them were their technical parameters: core diameter, refractive index profile, and numerical aperture (NA). First, there was a standard telecommunication gradient-index MMF with a 62.5/125 µm core/cladding diameter and a numerical aperture NA = 0.275 (GIF625, Thorlabs, Newton, NJ, USA)—named later in the paper as MMF62.5. The next one was a step-index MMF with a 105/125 µm core/cladding diameter and NA = 0.22 (FG105LCA, Thorlabs, Newton, NJ, USA)—named MMF105, and the last one was a step-index MMF with a 200/225 µm core/cladding diameter and NA = 0.39 (FT200EMT, Thorlabs, Newton, NJ, USA)—named MMF200. This selection allows testing of microtips with different shapes and sizes [3].

At the next stage of the study, the reflective properties of the proposed above monomer mixtures, light sources, and optical fibers were measured. To evaluate the effect of the amount of energy supplied in the manufacturing of a microtip, LASER 532 was used due to its maximum emitting optical power level of around 50 mW. Light from the laser was attenuated by a neutral density filter and then launched to the selected MMF. By changing the position of the filter, a proper amount of optical power was set. After closing the laser shutter, a drop of monomer was placed at the end of MMF. Next, opening the shutter for a certain time (exposure time) allowed the formation of a microtip when the monomer mixture was polymerized. Finally, the MMF with a microtip was tested by using the optical backscatter reflectometer OBR 4600 (Luna Technologies, Roanoke, VA, USA). This device measures an average return loss which shows changes in the optical backscattered signal. Return loss is calculated as the ratio of time-integrated amplitudes of selected impulses response to the amplitude of impulses response in the whole period as the following relation:

$$Return\;Loss=10\log\left(\overset{t_{2}}{\underset{t_{1}}{\sum }}\left|\tilde{h_{j}}\right|/\overset{T}{\underset{0}{\sum }}\left|\tilde{h_{j}}\right|\right)$$

where: *T*—time period, (*t_1_, t_2_*)—selected time period, $\tilde{h_{j}}=\mathrm{FFT}\;{\left\{H\left(\omega \right)\right\}}_{j}$ where $\mathrm{FFT}\;{\left\{H\left(\omega \right)\right\}}_{j}$—the Fast Fourier Transform of linear transfer function H(ω): $H\left(\omega \right)=\rho \left(\omega \right)e^{i\phi \left(\omega \right)}$. The above definition was adapted from OBR 4600 user guide and it is based on a measurement scheme of this device [34]. An uncertainty of ±0.16 dB based on deviation from the average result was determined. All tests were performed at a wavelength of 1550 nm. 

**Figure 1:**
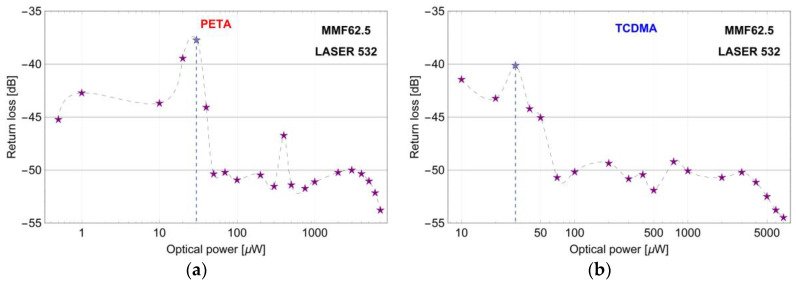
Return loss measured by OBR 4600 vs. optical power levels of LASER 532 used to manufacture different microtips based on the VIS mixture with monomers: (**a**) 3-functional pentaerythritol triacrylate (PETA) and (**b**) 2-functional tricyclo decanedimethanol diacrylate (TCDMA). Exposition time for all microtips equals 30 s. The blue dashed line indicates the optimal structure obtained during the manufacturing procedure.

In Figure 1, the return loss for different microtips manufactured at the end of MMF62.5 based on the VIS mixture with PETA and TCDMA was shown. 

All elements were manufactured with an optimal (from a mechanical point of view) exposition time equal to 30 s but with a different optical power level of LASER 532 which was within the range of 0.1–7000 μW. This power is used as a parameter for a micro-transducer identification in Figure 1. The return loss for these elements was measured by OBR 4600 working in a gain mode of the detection subsystem equal to 1.31 dB.

Taking into consideration the results from Figure 1, the micro-transducers have the maximum return loss when they were manufactured by an optical power of 30 μW, for both types of monomers. Assuming the return loss at the level of −45 dB, PETA based microtips have required optical properties for optical power levels from 0.5 μW to 40 μW (Figure 1a) and TCDMA based microtips from 10 μW to 50 μW (Figure 1b). The latter microtip started to form at the optical power of around 10 μW. The return loss of PETA-based microtip was higher by about 2dB than TCDMA microtip. 

Tests of UV LED and VIS LED sources were carried out first on MMF62.5. The manufactured microtips by using LEDs had a limited range of available optical power due to the coupling losses between LED output optical fiber (step-index with a 200 μm core) and MMF62.5. The maximum optical power levels for photopolymerization of around 70 μW for UV LED and 38 μW for VIS LED were reached. However, these optical power levels allow manufacturing microtips with acceptable reflective properties. 

As a summary of this research, parameters for optimal microtips manufactured at the end of MMF62.5 were shown in Table 1. Such elements have been obtained by a different light source with the same energy used for the mixture’s photopolymerization (optical power and exposition time equal to 30 μW and 30 s, respectively). PETA based microtips have base diameters similar for all tested light sources and slightly smaller than the core of the fibers used. However, TCDMA-based microtips have a sufficiently smaller base diameter for the LASER532 source in comparison to LED sources. This can be a result of a more uniform spatial distribution of LED intensity.

The same optimization steps were carried out for MMF105 and MMF200. Optimization of reflective properties of this type of microelements manufactured by using LASER532 on both MMF105 and MMF200 was not performed due to back reflection signals at the level of −70 dB. Therefore, optimization was performed only for microelements obtained by using UV and VIS LEDs. Both LEDs sources had as the output an optical fiber with a 200 μm core, therefore, for MMF105 and MMF200 a sufficiently higher optical power for photopolymerization was available. For these optical fibers and both tested LED sources, sufficient reflective properties of microtips were obtained also at an optical power of around 30 μW. Optimal base diameters of microtips manufactured by LED sources on MMF105 and MMF200 had diameters of around 105 μm and 200 μm, respectively.

**Figure 2:**
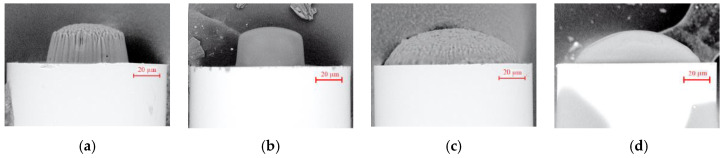
Scanning electron microscope images of optimal microtips prepared by: (**a**) LASER 532 on MMF62.5; (**b**) UV LED on MMF62.5; (**c**) LASER 532 on MMF105; (**d**) UV LED on MMF105. Manufacturing parameters: optical power—30 μW and exposition time—30 s.

In Figure 2, the examples of optimal microtips as the scanning electron microscope images prepared by using LASER 532 and UV LED on MMF62.5 and MMF105 were shown.

Both microtips prepared by LASER532 (Figure 2a,c) have rough surfaces due to specific multi-mode characteristics of the excited by a coherent light source MMF62.5 and MMF105 [3]. Output modal characteristics of the same optical fibers illuminated by a broadband source have Gauss-like intensity profiles, therefore, examples presented in (Figure 2b,d) have smooth surfaces even though UV or VIS LED were used [3]. 

**Figure 3:**
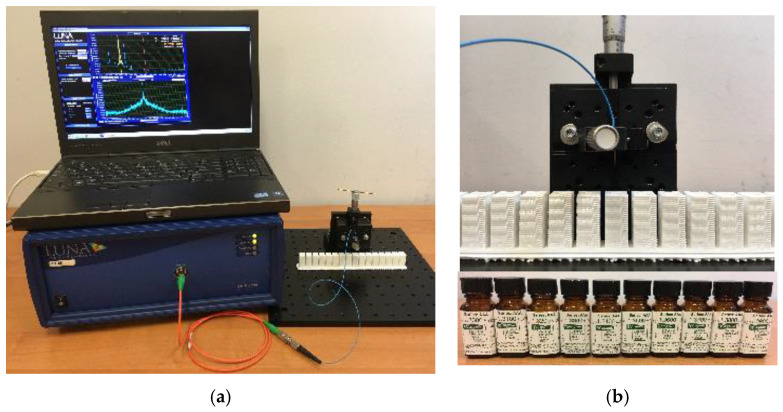
Experimental set-up for refractive index (RI) measurements by using the manufactured microtips—(**a**) and a manual stage with a fiber holder and a set of cuvettes with calibration liquids—(**b**).

In Figure 3, an experimental set-up used for RI measurements by using the manufactured micro-transducers is presented. 

In all measurements, the tested MMF with a microtip was connected at one end to OBR4600 (Figure 3a). The other end with a microtip was held vertically on a manual stage which facilitates immersion of the tested microtip in the selected liquid (Cargille Laboratories, Cedar Grove, NJ, USA) with a precisely known RI (Figure 3b). The set of cuvettes was filled with the calibration liquids. Before the microtip was immersed in another liquid, it was cleaned with alcohol and dried. The connection of MMF62.5 with OBR was relatively easy due to the use of a hybrid patch-cord. It had at one end a standard single-mode telecommunication optical fiber and at the other one—MMF62.5. However, for MMF105 and MMF200 splices with MMF62.5 pigtails had to be prepared by using a standard optical fiber fusion splicer (FSU-975, Ericsson, Stockholm, Sweden). 

**Figure 4:**
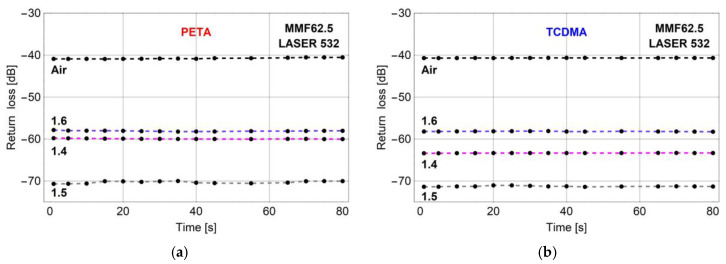
Return loss vs. time of the optimized microtips manufactured by using LASER 532 for monomers: (**a**) PETA and (**b**) TCDMA. Microtips surrounded by air—black dashed line, liquids with Figure 1. 4—magenta, 1.5—gray, 1.6—blue. Manufacturing parameters: optical power—30 μW and exposition time—30 s.

Tests of the manufactured micro-transducers began with time stability measurements of the signal reflected from the microtip kept in the air or immersed in the selected calibration liquids. In Figure 4, return loss as a function of time for microtips based on a VIS mixture with monomers: PETA (Figure 4a) and TCDMA (Figure 4b) was presented. 

Both presented plots show that time fluctuations of the measured back-reflected signal for microtips prepared with optimal parameters on MMF62.5 by LASER 532 are negligible. Return losses for micro-transducers made with PETA and TCDMA are kept signals levels, however, slightly differ only when immersed in a liquid with RI equal to 1.4. These measurements carried out for MMF62.5 with a microtip made with LASER 532 show that these optical elements even for a rough microtip surface (Figure 2a), return a stable optical signal.

## 3. Reflective Properties of a Micro-Transducer under RI Changes

In this part of the paper, the most important experimental results were presented. First of all, the selected MMFs without microtips were measured as a reference for comparisons of reflective properties of MMFs with microtips. Changes of RI of a material surrounding an optical fiber or a micro-transducer have changed the optical backscattered signal amplitude. The amplitude of this signal was minimal when the surrounding liquid RI was close to the RI of an MMF core or a tested microtip. The selected MMF or MMF with a microtip were immersed sequentially into liquids with various refractive indices within the range of 1.30–1.70. Figures presented below show changes in return loss vs. RI. Black dots with interpolation black dashed lines are the referenced return loss of optical fibers without microtips. Red and blue dots with approximation lines represent the experimental data obtained for microtips manufactured by using PETA and TCDMA monomers, respectively. Moreover, to evaluate the sensitivity of the reported sensor, the slopes of linear approximations of measurement data in selected RI regions were calculated. The sensitivity uncertainty was calculated as a mean value of single prediction errors at a 95% convenience level by using built-in procedures of *Wolfram Mathematica* software. The measurement data presented with the single predictions bands and approximation lines were plotted together for each sensor. Moreover, a standard R-squared value was used to evaluate the goodness of the linear fit model. The sensor resolution of RI was calculated as a multiplication of the inverse value of sensitivity and return loss resolution of 0.02 dB. 

In general, the microtip made at the end of all tested MMFs significantly changes the return loss characteristics. Light reflections are strongly disturbed when the flat surface of the cleaved MMF is replaced by the microtip curved polymer 3D structure. Additionally, the absorption of infrared light of polymer, optical fiber, and reference liquids has to be taken into account. 

**Figure 5:**
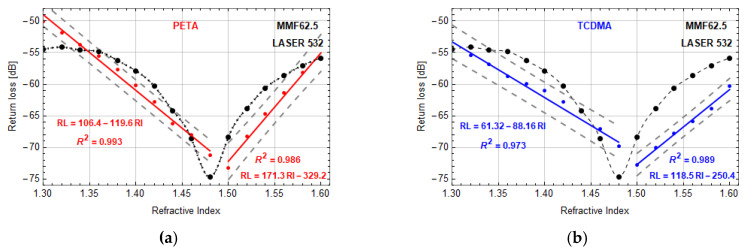
Return loss vs. RI—microtips manufactured by using LASER 532 on MMF62.5 based on monomers: (**a**) PETA—microtip size: 52 ± 1 µm base diameter, (**b**) TCDMA—microtip size: 34 ± 1 µm base diameter. Manufacturing parameters: optical power—30 μW and exposition time—30 s. Red and blue lines are linear approximations of measurement points with presented equations and coefficient of determination *R*^2^. Black dots are reference measurements of the return loss of MMF62.5 without microtip.

In Figure 5, the results for microtips on MMF62.5, manufactured by LASER 532, with PETA and TCDMA monomers were presented. In both plots, the background data of MMF62.5 without microtip (black dots with interpolation dashed lines) show that return loss changes are nonlinear with their minima at RI = 1.48 and the dynamic range of around 20 dB. The minimal value of RI is in good agreement with the core RI value for this optical fiber type. Two well defined RI ranges can be distinguished. The first one is from 1.30 to 1.48 of RI for which return loss decreases and the second one is from 1.48 to 1.60 for which return loss increases. 

Measurement data of a microtip with an optimal geometry based on PETA monomer (Figure 5a) show that the minimal value of return loss is shifted to 1.5 of RI and on both sides of it these data can be linearly approximated. The linear regression model was applied for a sensitivity evaluation for measurement points within two regions of RI, i.e., in the ranges of 1.30–1.48 and 1.5–1.6. In the first range, the sensor had a sensitivity of −119.6 ± 1.5 dB/RIU and its resolution was of 1.6 × 10^−4^ and in the second range of 171.3 ± 2.0 dB/RIU with a resolution of 1.1 × 10^−4^. A negative value of sensitivity means that return loss decreases with an increasing value of RI, whereas a positive value indicates an increase of return loss and RI. The change of a microtip material to a TCDMA monomer has brought a similar character of return loss changes. For this material, the optimal microtip has a base diameter of around 34 μm, and return loss is smaller than for the previous material. Despite this fact, return loss has kept its linear character in both RI regions, but its sensitivities and resolutions were of around −88.2 ± 2.2 dB/RIU, 2.3 × 10^−4^ and 118.5 ± 1.2 dB/RIU, 1.7 × 10^−4^ in respective RI regions. Moreover, for the VIS mixture with PETA monomer, the dynamic ranges were 23 dB in the RI range of 1.30–1.50, and 18 dB in the RI range of 1.50–1.60, while for the VIS mixture with TCDMA monomer, these dynamics were of 18 dB and 12 dB in the above-mentioned RI ranges. For all approximations in Figure 5, the coefficients of determination *R*^2^ are higher than 0.97. 

The next stage of the research focused on the application of mixtures polymerized by LED sources. In general, for optimal microtips manufactured by UV or VIS LED on MMF62.5 based on both monomers, the micro-transducers responses to RI changes were in good agreement with the data presented in Figure 5. However, for UV mixtures with TCDMA, the change of polymerization light sources increases the microtip base diameter what increases the return loss, as well. Visible differences of the surfaces of microtips manufactured by LASER 532 on MMF62.5 (Figure 2a) or LEDs (Figure 2b) do not have a significant impact on this sensor response. 

**Figure 6:**
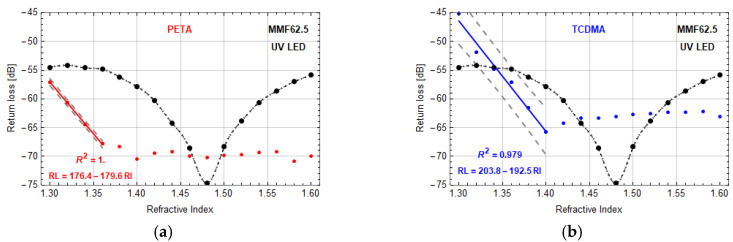
Return loss vs. RI—microtip manufactured by using UV LED on MMF62.5 based on polymers: (**a**) PETA, optical power 40 µW (microtip size: 61 ± 1 µm base diameter), (**b**) TCDMA, optical power 30 µW (microtip size: 58 ± 1 µm base diameter). Manufacturing parameter: exposition time—30 s. Red and blue lines are linear approximations of measurement points with presented equations and coefficient of determination *R*^2^. Black dots are reference measurements of the return loss of MMF62.5 without microtip.

In the next set of experiments, a possibility to increase these sensors’ sensitivity was tested. It was done by measurement of return loss changes of the microtips with larger than optimal base diameters. Both materials allow one to form microtips at the end MMF62.5 by using UV LED. The changes of return loss are significant due to a reduced range of RI changes for which data can be linearized. For PETA based microtip (Figure 6a), the RI range from 1.30 to 1.36 was approximated by linear regression. The sensitivity and resolution of this sensor were around −179.6 ± 0.3 dB/RIU and 1.1 × 10^−4^ with a dynamic range of 13 dB. Whereas for the TCDMA-based microtip (Figure 6b), the RI range was within 1.30–1.40. Sensor sensitivity and resolution were of around −192.5 ± 2.7dB/RIU, 1.0 × 10^−4^ with the dynamic range of 20 dB. Moreover, return loss characteristics for reference liquids with higher RIs were flattened. Comparison of data in both figures proves that the sensor sensitivity increase can be achieved by the microtip base diameter increase, however within a limited range of RI changes and decreased values of dynamic range and return loss. 

**Figure 7:**
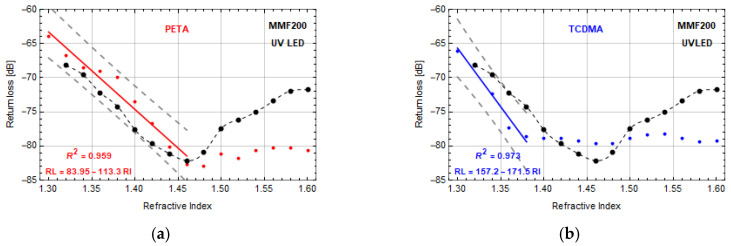
Return loss vs. RI—microtip manufactured by using UV LED on MMF200 based on polymer: (**a**) PETA—microtip size: 200 ± 1 µm base diameter, (**b**) TCDMA—microtip size: 196 ± 1 µm base diameter. Manufacturing parameters: optical power—500 μW and exposition time—30 s. Red and blue lines are linear approximations of measurement points with presented equations and coefficient of determination *R*^2^. Black dots are reference measurements of the return loss of MMF200 without microtip.

In the next step, MMF200 was used in tests. The surface roughness of a microtip manufactured by using LASER 532 resulted in a significant reduction of return loss. For this reason, no further research on this type of element was conducted. The reflective properties of microtips with the same geometry, manufactured by UV or VIS LED were similar for both materials used. Therefore, in Figure 7 the examples of return loss changes of micro-transducers on MMF200 made with PETA and TCDMA monomers by UV LED were presented. The change of an optical fiber to MMF200 resulted in forming a microtip covering almost the whole core of the optical fiber. Both return loss characteristics for this MMF were comparable to the data from Figure 6. Both tested sensors had linear response characteristics only within the range of 1.3–1.46 for PETA monomer (Figure 7a) and 1.30–1.38 for TCDMA one (Figure 7b). For PETA based monomer, this sensor in the mentioned range of RI had the sensitivity and resolution of around −113.3 ± 3 dB/RIU and 1.8 × 10^−4^ with the dynamic range of 19 dB. Moreover, these data are very close to the data of MMF200 without microtip at this range of RI. TCDMA-based sensor has a sensitivity and resolution of −172 ± 3 dB/RIU and 1.2 × 10^−4^ with the dynamic range of 20 dB in the mentioned above RI range. Comparing sensitivities of sensors based on MMF200 and MMF62.5, one can see that the latter has reached higher values of this parameter. 

**Figure 8:**
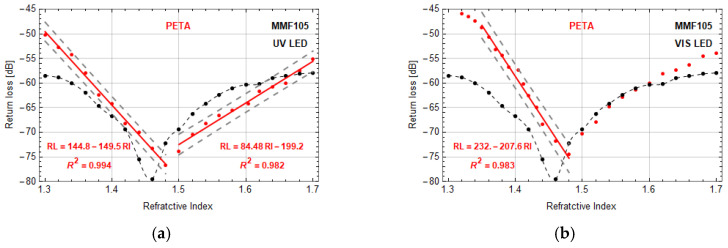
Return loss vs. RI—microtip manufactured on MMF105 based on PETA monomer by using: (**a**) UV LED, optical power 10 µW (microtip size: 84 ± 1 µm base diameter), (**b**) VIS LED, optical power 30 µW (microtip size: 105 ± 1 µm base diameter). Manufacturing parameter: exposition time—30 s. Red lines are linear approximations of measurement points with presented equations and coefficient of determination *R*^2^. Black dots are reference measurements of the return loss of MMF105 without microtip.

The last tested micro-transducers were manufactured on MMF105. Return loss changes of microtips based on PETA monomer were in good agreement with data obtained for TCDMA monomer. Moreover, microtips formed by both LEDs had similar return loss characteristics. Therefore, in Figure 8 the experimental results of sensors based on PETA monomer are presented. Similar to MMF62.5, the manufactured microtip on MMF105 also shifts the minimum return loss to a higher value of RI. This change is from 1.46 to 1.48. The return loss characteristics of a microtip with a base diameter smaller than the optimal one is presented in Figure 8a. For this sensor, two linear ranges of return loss are possible to distinguish. Sensitivity within the RI range of 1.30–1.48 is about −149 ± 1.6 dB/RIU with a dynamic range of about 26 dB. Within the RI range of 1.5–1.7, its sensitivity is 84.5 ± 1.7 dB/RIU and the dynamic range of 21 dB. For this microtip, the calculated resolutions are 1.3 × 10^−4^ and 2.4 × 10^−4^, respectively. These data are comparable with the data obtained for MMF62.5. For the optimal microtip in the RI range from 1.35 to 1.48, the achieved sensitivity and resolution are −207.6 ± 2.2 dB/RIU and 1.0 × 10^−4^ with the dynamic range of 32 dB (Figure 8b). In this case, both sensitivities, as well as dynamic range are the highest for all tested sensors.

## 4. Discussion

The extended optimization studies of polymer micro-transducers manufactured at the end of MMF allow the designing of an RI sensor with high sensitivity. The signal level reflected from such a micro-transducer depends on its geometry and material properties. Both of them are modified by the changing: monomer mixture composition, spectral characteristics of light, amount of delivered energy, and optical fiber type. For selecting two monomers, two types of mixtures polymerized by three different light sources and manufactured on three types of MMFs, the optical power was used as the key parameter of the optimization process. This parameter determines a microtip geometry and in this paper, it is reduced to a microtip base diameter, solely. However, optimal reflective properties of a microtip measured in the air do not fully represent the function of this element as a micro-transducer of an RI sensor. Taking into consideration sensitivity as the main parameter of the sensor calculated as a slope of linear changes of return loss, it is possible to describe the functionality of such micro-transducer. Microtip at the end of any of the selected MMF allows for changing a nonlinear curve of return loss characteristics of MMF without microtip into a linear one. These linear changes can be distinguished on both sides of minimum return loss with negative and positive slops at each of the two RI ranges. Nonlinear characteristics of the return loss of a simply cleaved MMF are the result of the Fresnel reflection at the boundary of optical fiber material and referenced liquid. Application of the polymer micro-transducer at the end of MMF causes a change in the return loss characteristics. Several factors contribute to this fact. First of all, most of the light propagated in the optical fiber is reflected on the microtip—liquid boundary which is evidenced by the shift of the characteristic minimum towards higher RI values. The next important issue is the reflection on the optical fiber end face—liquid interface, this is due to the fact that the optimal microtip did not cover the entire core of MMF. Moreover, optical attenuation of polymer, reference liquids, and optical fibers materials at the infrared region and reflection at the interface of optical fiber and micro-transducer have to be taken into consideration to fully explain return characteristics changes. Therefore, the sum of the above-mentioned effects results in the formation of linear characteristics of return loss.

Sensitivity analysis of this type of micro-transducer is based on the experimental results and it was defined as the slope of linear regressions in selected ranges of RI. The uncertainty of this parameter was assessed by using a mean value of single predicted errors calculated at the 95% confidence level. For microtips manufactured by LASER 532 on MMF62.5, the maximum sensor sensitivity is obtained for the microtip with optimal geometry. Increasing the microtip base diameter reduces sensitivity. However, for microtips polymerized by LED sources, the optimal size does not correspond to the highest sensitivity. In this case, the sensor sensitivity can be modified by changing the base diameter. By its increase, in relation to the optimal, an increase in sensitivity is obtained, but the RI range is narrowed to 1.30–1.36 and 1.30–1.40 for PETA and TCDMA monomers, respectively. The same results were observed for microtips manufactured on MMF105 and MMF200 by using LED sources. Increasing the base diameter has increased sensor sensitivity while narrowing the RI range. Sensitivities of these sensors based on both materials are comparable with the data presented in references [11,14,25,26,27,28].

Selected monomers allow building 3D polymer microelements for both UV and VIS sensitive mixtures. In the case of MMF62.5, the sensor with a microtip based on PETA monomer has a better sensitivity when the micro-transducer is manufactured by LESER532 while the sensor with TCDMA based monomer has a higher sensitivity when a microtip was enlarged when it was formed by UV LED. Both monomer mixtures applied to MMF105 and MMF200 allow to prepare microtip which works comparably. However, microtips made by LASER532 have formed rough-surfaced microelements and their reflective properties were unacceptable for RI changes measurements for both MMFs. Optimal microtips formed by LEDs on MMF200 covered fully the core of this fiber what has reduced RI measurement ranges of the sensors with microtips made of both monomers. Microtips on MMF105 allows for obtaining similar results for both monomers. The sensor with the optimal micro-transducer has the highest sensitivity of around −208 dB/RIU and a dynamic range of 32 dB.

The proposed RI sensor with a polymer micro-transducer has many advantages, such as easy and low-cost production, a wide range of operating temperatures, simple structure, and miniature size. Therefore, further research in this area will be carried out to improve the sensitivity and resolution of such type of optical fiber RI sensor.

## Figures and Tables

**Table 1:** The main parameters of microtips with optimal reflective properties, manufactured on MMF62.5 by means of different light sources (used optical power 30 μW and exposition time equal to 30 s).

Source	PETA	TCDMA
	Microtip Base Diameter [µm]	Microtip Base Diameter [µm]
LASER 532	50 ± 1	35 ± 1
UV LED	53 ± 1	50 ± 1
VIS LED	51 ± 1	50 ± 1
